# Dietary fat intake and gestational weight gain in relation to estradiol and progesterone plasma levels during pregnancy: a longitudinal study in Swedish women

**DOI:** 10.1186/1472-6874-9-10

**Published:** 2009-04-30

**Authors:** Marie Lof, Leena Hilakivi-Clarke, Sven Sandin S, Sonia de Assis, Wei Yu, Elisabete Weiderpass

**Affiliations:** 1 Department of Medical Epidemiology and Biostatistics, Karolinska Institute, Box 281, SE-171 77 Stockholm, Sweden; 2 Department of Oncology, Georgetown University, Washington, DC 20057, USA; 3 The Cancer Registry of Norway, Montebello, N-0310, Oslo, Norway; 4 Institute of Community Medicine, University of Tromsø, Breivika N-9037, Norway; 5 Department of Genetic Epidemiology, Folkhälsan Research Center, Samfundet Folkhalsan, Biomedicum Helsinki, Haartmansgatan 8, PB 63 (C312), Helsinki, FIN-00014 HU, Finland

## Abstract

**Background:**

Elevated pregnancy hormone levels, such as oestrogen and progesterone, may increase the risk of developing breast cancer both in mothers and offspring. However, the reasons for large inter-individual variations in estrogen and progesterone levels during pregnancy remain unknown. The objectives of this study were to investigate whether a) intakes of total dietary fat, types of fat (monounsaturated: MUFA, polyunsaturated: n-3 and n-6 PUFA, and saturated) and b) gestational weight gain are associated with estradiol and progesterone levels in plasma during pregnancy.

**Methods:**

We measured body weight as well as estradiol and progesterone in plasma among 226 healthy pregnant Swedish women on gestation weeks 12, 25 and 33. At the same time points, dietary intake of total fat and types of fat (MUFA, PUFA, SFA, n-3 and n-6 PUFA) were estimated using 3-day food diaries.

**Results:**

A large variation in estradiol and progesterone levels was evident.

Nulliparous women had 37%, 12% and 30% higher mean estradiol levels on gestation weeks 12, 25 and 33 compared to parous women (P = 0.008). No associations were found between dietary intake of total fat or fat subtypes (including n-3 PUFA and n-6 PUFA) and plasma estradiol or progesterone levels. Gestational weight gain was associated with progesterone levels (P = 0.03) but the effect was very small (20% increase in progesterone levels between gestational weeks 12 and 33 per kg body weight/week).

**Conclusion:**

No associations among gestational weight gain, maternal dietary fat intake (total or subtypes including n-3 PUFA and n-6 PUFA) and plasma estradiol levels were found. However, pregnancy progesterone levels correlated with weight gain during pregnancy. Identification of other possible determinants of pregnancy estradiol and progesterone levels, important for the development of breast cancer in both mothers and offspring, are needed.

## Background

Low parity and late age at first full-term birth are well-established risk factors for breast cancer in the mothers [1]. Recently it has also become evident that the environment *in utero *modifies the breast cancer risk in the female offspring. A large body of epidemiological evidence shows that a high birth weight increases breast cancer risk [2,3]. The biological mechanisms underlying these associations are not fully known, but variations in maternal hormone levels during pregnancy e.g. estrogens or progesterone have been proposed to play a role [1,4]. The estrogen-hypothesis is supported by findings showing that pregnant women [5] who haven taken diethylstilbestrol (a synthetic estrogen) exhibit increased breast cancer risk, and the findings from a nested-case control study in which women with higher pregnancy estrone levels during the third trimester had a two-fold incidence of breast cancer compared to controls [6]. Furthermore, several studies have reported positive associations between maternal estrogen levels and fetal size [7,8]. For progesterone, Hispanic women who are at lower risk of breast cancer than Caucasian women have been reported to have the lowest pregnancy levels of progesterone [9].

Pregnancy is characterised by a substantial increase in oestrogen and progesterone levels [10], and these levels also exhibit a marked inter-individual variability during pregnancy [11]. The reason for this inter-individual variability is unknown, but e.g. diet, particularly dietary fats, may be important. Earlier studies, using animal models, indicate that maternal consumption of a high fat diet containing high amount of n-6 polyunsaturated fatty acids (n-6 PUFA) statistically significantly increases pregnancy estradiol levels [12,13] as well as the incidence of mammary tumours in dams and offspring [12,13]. Such associations may also exist in humans, but available data are limited. So far only one study has been published reporting on the associations between maternal dietary intake of n-3 and n-6 PUFAs and pregnancy estradiol levels [14]. That study provided some evidence supporting the animal studies results, namely a positive association between PUFA and umbilical cord estriol levels as well as an inverse association between n-3 PUFA with umbilical cord estradiol levels among Japanese women [14]. Dietary intake was measured only once, on gestation week 29. No corresponding data for n-3 and n-6 PUFA in relation to maternal estradiol levels are available in Caucasian women who have higher breast cancer incidence.

The inter-individual variations of pregnancy estrogen and progesterone levels may also be explained by variations in weight gain during pregnancy. The adipose tissue acts as an endocrine and metabolic organ and thus influences the synthesis and bioavailability of estrogens. Both epidemiological and animal data suggest that women who gain excessive amounts of weight during their pregnancies have an increased breast cancer risk later in life [15,16]. However, longitudinal data regarding gestational weight gain in relation to pregnancy hormone levels are very limited. Two studies reporting no associations between total gestational weight gain and estriol [17,18] and estradiol levels [18] and a weak negative association for progesterone [18] are available, but none of these studies evaluated weight gain and pregnancy hormone levels during the different trimesters of pregnancy.

We present here results from a prospective cohort study in pregnant Swedish women whose intakes of a) total dietary fat, types of fat (monounsaturated: MUFA, polyunsaturated: n-3 and n-6 PUFA, saturated:SFA) and b) gestational weight gain were compared to plasma estradiol and progesterone levels on gestation weeks 12, 25 and 33.

## Methods

### Subjects

Two-hundred-ninety pregnant women were recruited from April 2000 through November 2003 before gestational week 12 at Solna maternity unit, Stockholm, as previously described [19]. These 290 women filled in a questionnaire on gestation week 12 covering health status, smoking and education. On gestation week 12, 25 and 33, their body weights were measured in light clothing without shoes on the scale (SECA delta, AJ Medical, Stockholm, Sweden) and a blood sample was drawn to estimate plasma levels of estradiol and progesterone. Furthermore, pregnant women completed 3-day food diaries on gestation weeks 12, 25 and 33. Infant birth characteristics were obtained from medical records. Sixteen women were lost to follow-up after the initial meeting and for the present analysis 48 were excluded due to twin pregnancy (n = 6) or because they had missing data on the baseline questionnaire (n = 7) or on one of the three measurements for body weight, dietary intake, estradiol or progesterone levels (n = 35). Thus, 226 women were included to the present study. Average body mass index (BMI), age, education, and parity of the 64 women who opted out of the study or were excluded were similar to the 226 participating women. The 226 women were healthy and had no history of hypertension, diabetes or thyroid problems. All women signed a written informed consent before inclusion in the study. The study was approved by the ethics committee at the Karolinska Institute (dnr 00-141).

### Dietary intake

The women recorded their food intake by means of food diaries during three consecutive days, including one weekend day. Portion sizes of food and drinks were weighed by the women themselves using a standard calibrated balance. The women received the food diaries and personal instructions on how to fill in the diaries from one trained midwife. Intake of energy, total fat, monounsaturated fat, saturated fat, polyunsaturated fat, n-3 PUFA and n-6 PUFA, carbohydrates and protein were calculated by linking reported food intake to the food composition table from the National food administration from 2004. Average values for the three days were used. Dietary intake of long chain n-3 PUFA was calculated as the sum of docosahexaenoic acid (DHA) and eicosapentaenoic acid (EPA). The calculations of energy and nutrients were conducted by M Löf who is a trained nutritionist. She contacted the women when she had problems to interpret the written information in the diaries.

### Estradiol and progesterone levels in the plasma

The plasma samples were stored at -80°C. Estradiol and progesterone levels were assessed using the Beckman Coulter (formerly DSL) – DSL-4400 estradiol double antibody kit and Siemens (formerly DPC) – TKPG2 kit, respectively. The antibodies used have high affinity for estradiol and progesterone, respectively, and low cross-reactivity to other naturally-occurring hormones. In the estradiol assay, the minimum detection limit is 10 pg/mL, and the coefficient of variation for intra-assay replicates is 5.2% and for inter-assay duplicates 10.6%. In the progesterone assay, the minimum detection limit is 0.10 ng/mL, and the coefficient of variation for intra-assay replicates is 4.1% and for inter-assay duplicates 6.4%. All plasma samples from each woman were analyzed in the same series and performed in duplicates.

### Statistical data analysis

To evaluate the study objectives a series of linear mixed effect models were fitted. The models utilized the within-subject measurements for the relation between estradiol or progesterone and weight gain (kg/week) and nutrients [total fat intake (g/day), SFA (g/day), MUFA (g(day), PUFA (g/day), n-3 PUFA (g/day), n-6 PUFA(g/day) and long n-3 PUFA (g/day)] at week 12, 25 and 33 while adjusting for baseline characteristics for the variables age at enrollment (years), BMI before pregnancy, (kg/m^2^), parity (number of children before index pregnancy), smoking (Yes/No), and education (2–3 years of secondary school or less, non-university degree or university degree). Total fat and subtypes of fat were energy adjusted using the residual method [20]. Since the estradiol and progesterone data was right skewed and with a few high outliers these variables were analyzed on the log scale.

Models were fitted for the analysis of the relation between estradiol as well as progesterone and a) intake of total or types of fat b) weight gain. Separate models were evaluated for fat intake and gestational weight gain since excessive gestational weight gains may result from a high dietary fat intake but also be the consequence of eating excessive amounts of other energy-yielding nutrients (protein or carbohydrates) or from a low physical activity. Additionally, we fitted models where we included both fat (total or subtypes) and weight gain. We also fitted models with SFA+MUFA+PUFA and n-3 PUFA+n-6PUFA, respectively. The fitness of the models fitted was evaluated with respect of normality and single gross outliers utilizing model residuals.

As an alternative approach for handling the within subjects variation when analysing the baseline variables the estradiol (or progesterone), total fat and fat subtypes were summarized by calculating the area under the estradiol (or progesterone), total fat and fat subtypes versus time curve (AUC). Division of AUC by 22 weeks produced a mean value for the whole period between gestation weeks 12 and 33 (mean area under the curve, MAUC). However, as the results obtained using this approach were very similar to the results from the models above we do not report results obtained using the MAUC estimates.

We also evaluated models allowing for different effects of the baseline characteristics at the different time points by including an interaction term between each baseline characteristic and time (gestational week 12, 25 and 33).

Statistical significance was considered at the two-sided p < 0.05 level. All statistical analyses were carried out using the SAS software procedure Mixed for repeated measurements, version 9.1.

## Results

The characteristics of the 226 subjects are shown in Table 1. The measured body weights and the reported dietary intake of total fat, SFA, MUFA, PUFA, n-3 PUFA and n-6 PUFA on gestation weeks 12, 25 and 33 are shown in Table 2.

**Table 1:** Characteristics of women in the study (n = 226)

Age (years), mean ± SD (range)	32 ± 4 (22–42)
Body weight before pregnancy (kg)	
mean ± SD (range)	64.9 ± 9.4 (46.0–96.0)
median (25^th^, 75^th ^percentile)	63.0 (58.0–68.0)
BMI before pregnancy (kg/m^2^)	
mean ± SD (range)	22.9 ± 3.0 (17.1–34.6)
median (25^th^, 75^th ^percentile)	22.2 (20.8–24.1)
Parity, n (%)	
0	150 (66%)
1	64 (28%)
2	12 (5%)
Education, n (%)	
Low (two to three years secondary school or less)	72 (32%)
Medium (non-university degree)	20 (9%)
High (university degree)	134 (59%)
Smoking during pregnancy, n (%)	
Yes	8 (4%)
No	218 (96%)
Birth weight of infant* (g),	
mean ± SD (range)	3630 ± 510 (2420–4950)
median (25^th^, 75^th ^percentile)	3630 (3330–3990)
Birth length of infant* (cm)	
mean ± SD (range)	51 ± 2 (44–57)
median (25^th^, 75^th ^percentile)	51 (50.0–52.0)

N, number of subjects; SD, standard deviation *51% boys, 49% girls

**Table 2:** Body weight and dietary fat intake at different stages of pregnancy (n = 226)*

	Gestational week 12	Gestational week 25	Gestational week 33
Body weight (kg)	66.6 ± 10.0	73.3 ± 10.1	77.7 ± 10.4
Total energy (kJ/day)	8710 ± 1720	9330 ± 1740	9310 ± 1760
Total fat (g/day)	73.2 ± 21.2	81.9 ± 21.2	81.3 ± 20.8
SFA (g/day)	31.2 ± 9.9	35.0 ± 10.0	35.3 ± 10.4
MUFA (g/day)	26.3 ± 8.4	29.6 ± 8.4	28.9 ± 8.0
PUFA (g/day)	10.5 ± 3.7	11.5 ± 4.1	11.2 ± 3.9
n-3 PUFA (g/day)	1.92 ± 0.76	2.07 ± 0.93	1.97 ± 0.77
Long n-3 PUFA (g/day)	0.27 ± 0.37	0.25 ± 0.34	0.20 ± 0.27
n-6 PUFA (g/day)	8.44 ± 3.17	9.27 ± 3.49	9.07 ± 3.41
n-6 PUFA/n-3 PUFA	4.64 ± 1.36	4.92 ± 3.23	4.85 ± 1.46

*Values are means ± SD

**Figure 1:**
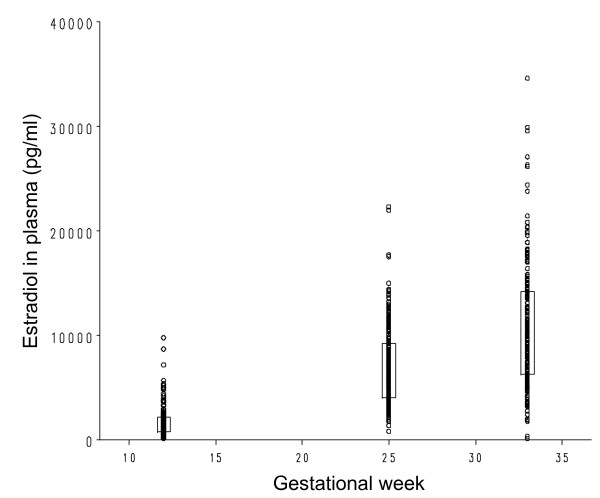
**Estradiol in plasma (pg/ml) on gestation weeks 12, 25 and 33**. Boxes are drawn between the 25^th ^and 75^th ^percentiles with the median rate marked as a straight line. Estradiol in plasma for the 226 individual women is shown as unfilled circles.

**Figure 2:**
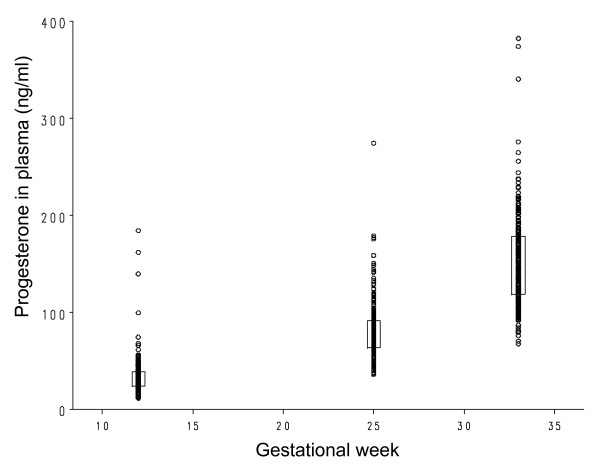
**Progesterone in plasma (ng/ml) on gestation weeks 12, 25 and 33**. Boxes are drawn between the 25^th ^and 75^th ^percentiles with the median rate marked as a straight line. Progesterone in plasma for the 226 individual women is shown as unfilled circles.

As expected the estradiol as well as the progesterone levels increased substantially throughout pregnancy (Figures 1 and 2). The mean estradiol levels were 600% higher on gestation week 25 compared to gestation week 12 (P < 0.0001) and 150% higher on gestational week 33 compared to gestational week 25 (P < 0.0001). The corresponding increases in progesterone were 240% from gestational week 12 to gestational week 25 (P < 0.0001) and 190% from gestational weeks 25 to gestational week 33 (P < 0.0001). Furthermore, also shown in Figure 1, a large variation in estradiol and progesterone levels were evident.

The estradiol levels were also affected by parity. The mean estradiol levels for nulliparous women were 1826, 7327 and 11201 pg/ml on gestation weeks 12, 25 and 33, respectively, and these figures were statistically significantly higher than the corresponding values for parous women: 1346, 6553 and 9532 pg/ml (P = 0.008). Progesterone levels were not affected by parity.

The associations between estradiol levels and weight gain, total fat intake and fat subtypes are shown in Table 3. No statistically significant association between weight gain or estradiol levels was found in the univariate model or when we adjusted for maternal age, BMI before pregnancy, smoking, parity and education. Correspondingly, no statistically significant associations between estradiol levels and total fat, SFA, PUFA, MUFA, n-3 PUFA, long n-6 PUFA or n-6 PUFA were found in our univariate or multivariable models. Similar results were obtained when we fitted several types of fat in the same model (SFA+MUFA+PUFA and n-3 PUFA+n-6PUFA). Furthermore, similar results were obtained when we fitted gestational weight gain and fat (total or subtypes) intake in the same model (data not shown).

**Table 3:** Weight gain and dietary fat (total and subtypes) in relation to plasma estradiol levels.

	Univariate*		Multivariable#	
	Percentage change in estradiol (95% CI)^§^	P	Percentage change in estradiol (95% CI)^§^	P
Weight gain (per 1 kg/week)	2.0 (-14, 18)	0.808	1.8 (-14, 18)	0.824
Total fat (per 10 g/day)	0.04 (-3.7, 3.8)	0.983	0.53 (-3.1, 4.3)	0.779
MUFA (per 10 g/day)	0.81 (-7.1, 9.4)	0.845	1.9 (-6.1,10.6)	0.651
PUFA (per 5 g/day)	0.97 (-4.9, 7.2)	0.753	1.2 (-4.7, 7.5)	0.699
SFA (per 10 g/day)	-2.6 (-9.0, 4.2)	0.450	-1.2 (-8.3, 5.2)	0.606
n-3 PUFA (per 1 g/day)	-3.0 (-8.0, 2.2)	0.253	-3.1(-8.1, 2.2)	0.249
n-6 PUFA(per 1 g/day)	-0.21 (-1.9, 1.5)	0.806	-0.18 (-1.8, 1.5)	0.834
Long n-3 PUFA (per 0.1 g/day)	-0.19 (-1.6, 1.3)	0.796	-0.25(-1.7, 1.2)	0.733
n-6 PUFA/n-3PUFA (per 0.5)	0.25(-0.53,1.0)	0.532	0.33(-0.45,1.1)	0.417

*Time of measurement (week 12, 25 or 33) was included as a variable in the model

^#^Adjusted for the following maternal characteristics: smoking, education, parity, age and BMI before pregnancy and time of measurement (gestational week 12, 25 or 33).

^§^This was computed as (10^×^-1) × 100 where × is coefficient obtained from the multiple linear analyses.

The corresponding associations between progesterone levels and weight gain, total fat intake and fat subtypes are shown in Table 4. A statistically significant association between gestational weight gain and progesterone levels was found (P = 0.03) but the effect was small: a 1 kg/week increase on gestation weight gain was associated with a 20% increase in progesterone levels between gestational weeks 12 and 33. No statistically significant associations between progesterone levels and total fat, SFA, PUFA, MUFA, n-3 PUFA, long n-6 PUFA or n-6 PUFA were found in any of our univariate or multivariable models irrespectively if we fitted the fat types in separate models or combined several of them in the same model (SFA+MUFA+PUFA and n-3 PUFA+n-6 PUFA).). Similar results were obtained when we fitted gestational weight gain and fat (total or subtypes) intake in the same model (data not shown).

**Table 4:** Weight gain and dietary fat (total and subtypes) versus plasma progesterone levels.

	Univariate*		Multivariable#	
	Percentage change in estradiol (95% CI)^§^	P	Percentage change in estradiol (95% CI)^§^	P
Weight gain (per 1 kg/week)	22 (20, 42)	0.029	22 (20, 42)	0.030
Total fat (per 10 g/day)	-3.3 (-7.5, 1.2)	0.149	-3.0 (-7.3, 1.5)	0.190
MUFA (per 10 g/day)	-4.1 (-13.2, 6.0)	0.415	-3.4(-12.6,6.9)	0.505
PUFA (per 5 g/day)	-1.6 (-8.5, 5.9)	0.673	-1.4 (-8.5, 6.1)	0.700
SFA (per 10 g/day)	-4.7 (-12.4, 3.5)	0.245	-4.3 (-12.0, 4.1)	0.307
n-3 PUFA (per 1 g/day)	-2.8 (-9.0, 3.9)	0.411	-2.8(-9.1, 3.8)	0.395
n-6 PUFA(per 1 g/day)	-0.21 (-1.9, 1.5)	0.806	-0.18 (-1.8, 1.5)	0.834
Long n-3 PUFA (per 0.1 g/day)	-0.19 (-1.6, 1.3)	0.796	-0.25(-1.7, 1.2)	0.733
n-6 PUFA/n-3 PUFA (per 0.5)	0.16(-0.85,1.2)	0.756	0.17(-0.8,1.2)	0.739

*Time of measurement (week 12, 25 or 33) was included as a variable in the model

^#^Adjusted for the following maternal characteristics: smoking, education, parity, age and BMI before pregnancy and time of measurement (gestational week 12, 25 or 33).

^§^This was computed as (10^×^-1) × 100 where × is coefficient obtained from the multiple linear

analyses.

We also tested if total energy intake or intake of the other energy-yielding nutrients: carbohydrate and protein were associated with estradiol or progesterone plasma levels. However, none of these variables were associated with estradiol or progesterone levels (data not shown).

We found no evidence for an interaction between any of the baseline characteristics and time of measurement (i.e. pregnancy week when the samples were taken) and levels of estradiol and progesterone (data not shown).

## Discussion

We report here results from the first study which have measured maternal intakes of dietary fat (including n-3 PUFA and n-6 PUFA) using food diaries in all trimesters in relation to maternal plasma estradiol and progesterone levels in a group of Caucasian women. No association between dietary intakes of total fat or fat subtypes (including n-3 PUFA and n-6 PUFA) and plasma estradiol or progesterone levels were found. Gestational weight gain was not associated with plasma estradiol levels, while a positive association was found for progesterone.

To our knowledge this is the first study to evaluate n-3 PUFA and n-6 PUFA in relation to maternal progesterone levels. Further, only one earlier study has investigated the impact of n-3 PUFA and n-6 PUFA on pregnancy estradiol levels. In 189 Japanese pregnant women dietary intake was measured on gestation week 29 using a food diary and pregnancy estrogen levels were measured on gestation week 10 and 29. No association was found between maternal dietary intakes of n-3 PUFA or n-6 PUFA and serum estradiol levels. However, a positive association between n-6 PUFA and umbilical cord estriol levels as well as an inverse association between n-3 PUFA with umbilical cord estradiol levels was found [14]. In our study, conducted in a Caucasian population with a high risk of breast cancer, did not provide any support for an opposing effect of n-3 and n-6 PUFA on pregnancy estradiol levels. The somewhat inconsistent results may be due to several reasons. First, the associations for n-3 and n-6 PUFA and estradiol in Japanese women were only detected in umbilical cord, and we measured estradiol in circulating plasma. Second, the populations differ in several respects. Estradiol levels were higher in the Japanese women, which in agreement with earlier reports comparing estradiol levels in Asian and Caucasian pregnant women [9]. Furthermore, the dietary intake of long n-3 PUFA was twice as high in the Japanese women.

Three earlier studies have evaluated the associations between pregnancy estradiol levels and maternal dietary intakes of total fat, MUFA, PUFA and SFA [14,18,21], and one of these studies also investigated the corresponding associations for progesterone [18]. Their results are consistent with our findings. The first one was conducted in 141 pregnant Greek women and the authors found no association between pregnancy estradiol levels and maternal dietary fat (total, MUFA, PUFA or SFA), measured using a food frequency questionnaire (FFQ) in pregnancy week 26 [21]. The second study which was conducted in 270 American women, did not find any association between pregnancy estradiol or progesterone levels and maternal dietary fat (total, MUFA, PUFA or SFA) estimated using a FFQ either on gestation weeks 16 and 27 [18]. The third study is the Japanese study referred to above and they also reported no association between total fat, MUFA, PUFA or SFA and estradiol levels on gestation week 29 [14].

To our knowledge no other study has evaluated the impact of gestational weight gain using repeated measurements of body weight during pregnancy on maternal plasma estradiol or progesterone levels. Still, in accordance to our findings, Lagiou et al reported no effect of total gestational weight gain on estradiol levels on gestation week 16 and 27 in a group of American women [18], and a very small effect of progesterone [18]. Furthermore, a recent study reported no association between total pregnancy weight gain and maternal umbilical cord estrogen levels [22]. Thus, although pregnancy weight gain has been linked to an increased breast cancer risk [15,16] the limited available data does not support a major role for maternal estradiol levels for this association. However, pregnancy progesterone levels were positively associated with weight gain during pregnancy. Still, this finding should be confirmed in other populations since we made multiple comparisons and our finding may be due to chance. Our animal studies indicate that other hormones e.g. leptin may be interesting in this context [15].

Two earlier studies have reported a positive associate between maternal alcohol intake and estradiol levels [14,23], while another study reported no association [24]. We did not adjust for alcoholic beverages in our study, since none of our women reported that they drank alcohol during pregnancy.

The strengths of our study are the prospective design and our repeated measurements of estradiol and progesterone levels and dietary fat intake from all three trimesters. Furthermore, in comparison to earlier studies in Caucasian women [18,21] we used food diaries to assess dietary fat intake. The food diaries may be advantageous for our purpose since they are developed to capture "current diet" compared to food frequency questionnaires which often estimate "usual diet" i.e. dietary habits during a longer time period, for instance one year before the dietary assessment. Body weight was also measured during standardised conditions. Limitations are that we cannot exclude some misclassification of dietary fat intake due to the fact that dietary intake was self-reported. Still such errors are likely not associated with estradiol or progesterone levels. Furthermore, we only measured the pregnancy hormone estradiol, and thus we do not know if our associations described are similar for the other estrogens such as estriol and estrone.

Our women were not randomly recruited. However, we have earlier concluded that this cohort is likely not very different regarding characteristics such as gestational weight gain and infant characteristics from other healthy pregnant women in Sweden and other Western countries [19]. The magnitudes of the increase in pregnancy estradiol and progesterone levels were also similar to earlier reports [10], and in our study nulliparous women had higher estradiol levels than parous women which is in agreement with findings from other populations [25]. Furthermore, our women's diet consisted of 31%, 11%, 4.5%, 13%, 1% and 3.5% of energy from total fat, MUFA, PUFA, SFA, n-3 PUFA and n-6 PUFA, respectively, and these figures agree very well with a large Swedish cohort of 49 000 pre-menopausal women for total fat, MUFA, PUFA, and SFA [26] as well as for n-3 PUFA and n-6 PUFA (unpublished data).

## Conclusion

We found no associations between gestational weight gain, maternal dietary fat (total or subtypes including n-3 PUFA and n-6 PUFA) and plasma estradiol levels. Plasma progesterone levels correlated with weight gain during pregnancy. Despite earlier animal findings [12,13], current knowledge in humans does not provide any strong evidence for an association between pregnancy dietary fat and pregnancy plasma estradiol levels. Identification of other determinants of pregnancy estradiol and progesterone levels, which have been proposed to be important for the development of breast cancer in both mothers and offspring, are needed.

## Competing interests

The authors declare that they have no competing interests.

## Authors' contributions

EW was responsible for the recruitment and data collection, while LHC was responsible for the analyses of estradiol and progesterone in plasma. ML processed all food diaries and calculated intake of energy and nutrients, prepared a database with all variables as well as performed the data analyses (in collaboration with SS who was responsible for the statistical analyses). SA and WY conducted the hormone assays. ML prepared the manuscript which was subsequently reviewed by LHC, SS and EW. All authors have approved the final manuscript.

## Pre-publication history

The pre-publication history for this paper can be accessed here:


